# Multiscale Engineered Heterogeneous Hydrogel Composites for Digital Light Processing 3D Printing

**DOI:** 10.1021/acsami.5c09635

**Published:** 2025-09-08

**Authors:** Yuang Zhang, Ryan Davis, Saptarshi Biswas, Sarah E. Miller, Syed Raza Ur Rehman, Gene T. Felix, Akhilesh K. Gaharwar

**Affiliations:** † Department of Materials Science and Engineering, College of Engineering, 2655 Texas A&M University, College Station, Texas 77843, United States; ‡ Department of Biomedical Engineering, College of Engineering, Texas A&M University, College Station, Texas 77843, United States; § Interdisciplinary Program in Genetics, Texas A&M University, College Station, Texas 77843, United States; ∥ Center for Remote Health Technologies and Systems, Texas A&M University, College Station, Texas 77843, United States

**Keywords:** biomaterials, digital light processing (DLP) 3D printing, hydrogel composite, microgels, regenerative medicine

## Abstract

Hydrogel-based bioinks are widely adopted in digital light processing (DLP) 3D printing. Modulating their mechanical properties is especially beneficial in biomedical applications, such as directing cell activity toward tissue regeneration and healing. However, in both monolithic and granular hydrogels, the tunability of mechanical properties is limited to parameters such as cross-linking or packing density. Herein, we present a bioink platform with multiscale heterogeneity for DLP printing, fabricated by incorporating microgels within a cross-linked polymer matrix to form a mechanically tunable heterogeneous hydrogel composite. The properties of the separate components as well as their interactions can be efficiently tailored from both chemical and physical perspectives, enabling control across both nano and micro scales. Monodisperse, spherical gelatin methacryloyl (GelMA) microgels with a stiffness that can be tuned through polymer concentration or cross-link density are fabricated by a high-throughput microfluidic device. Microgels that have been precross-linked through chemical or physical methods are then embedded in a continuous GelMA matrix, where they influence the biomechanical and biochemical characteristics of composites through particle density and encapsulation of cells. Modulation of microgel volume and selecting different printing parameters enables tailoring of the composite compressive modulus across a range of 29 to 244 kPa. Using this composite hydrogel platform as a DLP ink allows for the fabrication of complex 3D structures with macroscale heterogeneity, providing the potential to mimic tissue- and organ-level complexity. This study presents a unique approach to designing heterogeneous hydrogel composites with tunable properties at the nano-, micro-, and macro-scales, and introduces a highly modular hydrogel platform for DLP 3D printing.

## Graphical abstract

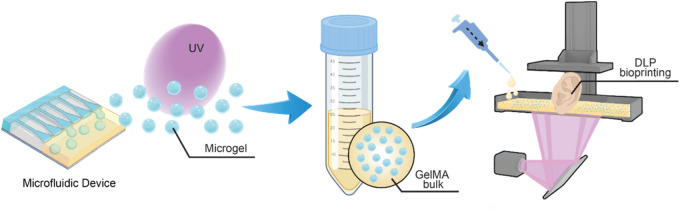

## 1 Introduction

Multiscale heterogeneity is present in almost every organ in the human body, reflected in the complex combination of biomolecules, cell populations, and structural architectures in tissues.^1^ From soft tissues such as the brain and liver to elastic and rigid tissues like cartilage and bone, compartmental heterogeneity in both biological and mechanical levels plays a vital role in maintaining appropriate organ functions.^1–7^ Given the inherent complexities of native tissues, it is challenging or even impractical to construct their functional heterogeneity using a single material, as such variations in microenvironment cannot be sufficiently reproduced by single-material processes.^8–10^

Previous studies have used multiple methods to fabricate hydrogels with mechanical heterogeneity. In traditional monolithic hydrogels, this is typically achieved through varying polymer concentrations, which ultimately determines the maximum cross-link density.^11^ Additionally, the mechanical properties of hydrogels can be altered through the introduction of strong hydrogen bonds,^12–14^ crystallization,^15,16^ or ionic interactions,^17–19^ to strengthen the cross-linking between polymer chains. While these approaches may efficiently alter the mechanical characteristics of hydrogels, the lack of structural heterogeneity is not addressed. Given the complex, dynamic nature of biological processes, heterogeneity is desired in hydrogel-based bioinks to better replicate natural phenomena. Traditional monolithic hydrogels are limited by their single-component nature, which hinders customization beyond the nanoscale.

Additive manufacturing, the process of building three-dimensional (3D) structures through a layer-by-layer method, has been applied in a wide range of biomedical applications.^20–23^ Macroscale heterogeneity can be achieved through this technology. Previous studies have demonstrated that 3D printing can be applied to fabricate customizable heterogeneous structures.^24–28^ For example, extrusion-based single-nozzle or multinozzle printing allows the use of different materials and can successfully build constructs with controlled heterogeneity.^8,29–31^ However, its inherent shear forces and relatively modest performance in small-scale fabrication remain key drawbacks, given the need for cell-laden and high-resolution printing in biomedical applications.^32–35^ Volumetric printing serves as another option for fabricating heterogeneous constructs. Its unique layer-less cross-linking mechanism enables the rapid printing of large constructs and allows the successful fabrication of heterogeneous constructs in a core–shell manner.^36–38^ Similar to extrusion printing, volumetric printing also has inherent limitations associated with its printing mechanism. For instance, it requires a large volume of resin to remain in the ink reservoir during printing.^39,40^ In addition, compared with other 3D printing technologies, the availability of commercially accessible volumetric printers remains limited at present.^41^

Digital light processing (DLP) 3D printing has emerged as a widespread additive manufacturing modality, recognized for its design flexibility, fast building speed, and high resolution for reproducing intricate 3D details.^42–45^ As a technology based on high-precision projecting light, DLP offers high printing accuracy and high efficiency.^46^ The working conditions in DLP are more conducive to cell viability due to reduced forces applied to the bioink. Due to its bottom-up printing mechanism, DLP enables the printed structure to gradually lift away from the ink reservoir, thereby reducing the required volume of bioink. Hydrogels are promising bioink candidates for DLP 3D printing due to their robust tunability, capacity to mimic natural tissue matrices, and versatility, as they provide a blank slate that can be modulated in several ways to achieve specific bioactivities and therapeutic effects.^47,48^ Modular mechanical properties are especially advantageous for tissue engineering, as these materials can be tailored to affect various cellular functions, such as migration,^49,50^ proliferation,^51^ or differentiation.^52–54^ Appropriate stiffness is required in tissue scaffolds to result in adequate structural and functional tissue restoration.^55,56^ Additionally, mechanical properties regulate the swelling and biodegradation of hydrogels, affecting their performance as drug delivery vehicles.^57,58^ Hence, the development of tunable hydrogel-based DLP inks is essential to meet the multifaceted requirements associated with bioprinting.

Recently, aqueous two-phase emulsion-based DLP bioinks have been developed to introduce microscale heterogeneity by forming microgel assemblies within structures during printing. However, these methods inherently produce random, uncontrolled microstructures due to the stochastic nature of emulsion droplet formation, limiting precise spatial control.^59^ While hybrid printing systems integrating DLP with direct ink writing have demonstrated impressive versatility in constructing heterogeneous, multifunctional structures, these systems rely on customized setups that are not commercially available.^60^ This limits accessibility and widespread use in standard bioengineering or industrial research environments.

Dual-component hydrogel composites, consisting of hydrogel microparticles embedded within a continuous matrix, provide a promising approach for developing multifunctional hydrogel bioink platforms with controlled heterogeneity. The presence of microgels brings structural heterogeneity to the system as they can serve as a separate compartment that is physically and functionally independent of the surrounding matrix.^61^ Although other types of particles, such as calcium-based,^62^ magnetic,^63^ or sacrificial particles,^64^ can be incorporated to influence matrix properties and introduce additional functions, they often lack the same level of versatility as microgels. In contrast, microgels offer enhanced versatility by serving as carriers capable of encapsulating drugs,^65–67^ cells,^68,69^ and enzymes,^70,71^ while simultaneously affecting the mechanical behavior of the matrix. The mechanical properties of the composite can be readily modulated by controlling the volume ratio of microgel to matrix.^72–74^ Incorporating microgels within a polymer matrix to form a composite bioink for DLP makes it feasible to develop hydrogel composites with tunable, multiscale mechanical heterogeneity. Ultimately, the composite has the potential to serve as a highly customizable platform for the development of multifunctional hydrogel systems.

Herein, we developed a hydrogel composite platform for DLP bioprinting by integrating precross-linked microgels within a bioink precursor. We first prepared microgels using a microfluidic device and combined them with a bioink precursor at different volume ratios to form heterogeneous composite bioinks. Subsequently, we explored engineering nanoscale heterogeneity of the composites by varying polymer concentrations in microgels and bulk, photoabsorber concentration, and UV exposure, and how this affected the overall stiffness. Next, the effects of modulating microscale interactions between the matrix and the microgels were investigated. Finally, to demonstrate and enhance its versatility at the macroscale, the hydrogel composite was employed in DLP printing to fabricate different biological 3D constructs.

## 2 Materials and Methods

### 2.1 GelMA Synthesis

GelMA synthesis was adapted from previously described protocols.^75,76^ Briefly, 10 g of gelatin (Type A, 300 bloom) was dissolved in 100 mL of DI water and heated to 50 °C before adjusting the pH to 8.5. Next, 8 mL of methacrylic anhydride (MA) was slowly added to the reaction mixture and allowed to stir for 3 h. The product was then centrifuged to remove unreacted MA, followed by dialysis for 3 days in DI water, frozen at −80 °C and lyophilized for 5 days. Lyophilized GelMA was dissolved in deuterium oxide (D_2_O) at a concentration of 15 mg/mL and analyzed using ^1^H NMR (Avance Neo 400) to determine the degree of methacrylate substitution.

### 2.2 Fabrication of Microgels and Heterogeneous Hydrogel Composites

Microfluidic devices were first fabricated using soft lithography to fabricate microgels. Photolithography was used to fabricate master molds on a 4-in. silicon wafer using a negative photoresistor (SU8 2000, MicroChem). Microfluidic devices were molded from master molds by pouring degassed poly­(dimethylsiloxane) (PDMS) (Sylgard 184, Dow, elastomer: curing agent = 10:1) and curing at 80 °C for 2 h. PDMS devices were then plasma-treated and adhered to a glass slide. Gel precursor solutions consisted of GelMA and lithium phenyl-2,4,6-trimethylbenzoylphosphinate (LAP) as a cross-linker. For visualization, 1 mg/mL of rhodamine-loaded Laponite was added to the precursor. Microgel droplets were generated at 100 vertical step junctions where the oil phase broke the stream of the precursor into spherical droplets. Syringe pumps were used to control the volumetric flow rates of each input stream. The oil flow rate: precursor flow rate ratio was maintained at 2:1, and the droplet generation speed was 1.13 × 10^6^/h. The generated droplets were photo-cross-linked into microgels downstream in the outlet tubing by 365 nm UV light at 30 mW/cm^2^ for 2 min to ensure full cross-linking. To form physically cross-linked microgels, droplets were generated and collected without UV exposure, followed by cooling at 4 °C overnight. Microgel size and polydispersity were characterized using fluorescence images analyzed in ImageJ.

To form heterogeneous hydrogel composites, microgels were mixed at varying volume ratios with a bioink precursor containing GelMA, LAP, and tartrazine prior to UV cross-linking. Confocal microscopy (Olympus FV1000) was performed to visualize the distribution of microgels within composite structures based on varying μgel/bulk volume ratios. Rhodamine-isothiocyanate dextran was added to the microgel precursor, while the bulk was left unmodified. Scanning electron microscopy (SEM) was also performed to evaluate hydrogel composites. Samples were prepared as described above, sectioned into thin slices, and sputter coated with platinum–palladium (Sputter Coater 208 HR, Cressington U.K.). The structure and surface topography of composites with varying μgel/bulk volume ratios were observed using a 5 kV electron beam (JSM-7500F, JEOL, Japan).

### 2.3 Cell Encapsulation in Photo-Cross-Linked Microgels

#### 2.3.1 Cell Culture

After obtaining human dermal fibroblast cells (HDFs) (ATCC) from multiple donors, the cells were cultured in an aseptic condition in 5% CO_2_ at 37 °C with Dulbecco-modified eagle medium (DMEM) high glucose (Cytiva-Hyclone), supplemented with 1% penicillin/streptomycin (100 U/100 μg/mL; Gibco). Human mesenchymal stem cells (hMSCs) (ATCC) were also obtained from multiple donors and cultured in previously described conditions with α-Minimum Essential Medium (α-MEM) (Cytiva-Hyclone), supplemented 1% penicillin/streptomycin (100 U/100 μg/mL; Gibco). After every 2 days, the media was changed with fresh media, and after obtaining 80% confluency, cells were passaged using 0.5% trypsin-EDTA (Gibco) at approximately 75,000 cells/cm^2^ for expansion.

#### 2.3.2 Fabrication of Cell-Laden Microgels

To form photo-cross-linked, cell-laden microgels, DMEM was used as a solvent to prepare the precursor, and HDFs were dispersed in the precursor with a final cell density of 5 × 10^5^ cells/ml in 7.5% GelMA. The generated droplets were photo-cross-linked using the previously specified UV intensity and exposure time. As the temperature must be maintained within a cell-friendly range and cell density may vary, the fluidity of cell-laden precursors could be compromised, which affects the monodispersity of the droplets. Finally, the cell-laden microgels were collected by centrifugation (500*g* for 10 min), redispersed in the medium, and transferred to a 96 well-plate containing DMEM and incubated at 37 °C for 1, 3, 5, and 7 days.

The cytocompatibility of the cell-encapsulated microgels was analyzed using the Alamar Blue assay. The assay was performed on days 1, 3, 5, and 7 according to the manufacturer’s protocol. Following incubation, the reading was taken at 570 and 600 nm with a plate reader (Tecan Group Ltd., Tecan Infinite 200Pro M Plex, Switzerland). The percent reduction was calculated after each time point, with the values on days 3, 5, and 7 normalized by the values obtained on day 1.

#### 2.3.3 Fabrication of Cell-Laden Composites

hMSCs were first stained using CellTracker fluorescent probes (Green-CMFDA, Invitrogen) according to the manufacturer’s instructions. hMSC-laden microgels were fabricated and collected following the previously described method using a precursor with a final cell density of 1.8 × 10^6^ cells/ml in 7.5% GelMA. To prepare composite bioink, hMSC-laden microgels were mixed with a 12.5% GelMA bulk. Subsequently, hMSC-laden hydrogel composites with a 10 mm diameter and 2.5 mm thickness were fabricated using DLP and transferred to a 24 well-plate containing α-MEM and incubated at 37 °C for 1, 3, and 5 days. At each time point, the brightfield and fluorescence images of hMSC-laden hydrogel composites were acquired with a microscope at 10× magnification (BioTek Lionheart LX, Agilent).

### 2.4 Mechanical Characterization

Hydrogel composites were fabricated using DLP and cut using a 6 mm biopsy punch to form 3 mm-thick cylindrical samples. Mechanical testing using an ADMET eXpert 7600 system (ADMET, Inc., Norwood, MA) with an attached load cell of 50 lb was performed to determine composites’ compressive moduli, failure stresses, and failure strains. Samples were compressed to 60% strain at a rate of 1 mm/min, and compressive moduli were calculated as the slope of the linear region of the loading curve.

### 2.5 Rheological Characterization

Preprint fluidity and gelation kinetics of hydrogel composites were assessed using an oscillatory stress-controlled rheometer (Discovery HR-2, TA Instruments) with an 8 mm parallel plate geometry and a 200 μm gap. Temperature sweeps from 5 to 45 °C were performed to investigate the viscosity of different ink formulations at printing temperatures. Time sweeps with delayed UV exposure were performed to assess the changes in complex moduli as composites were allowed to cross-link fully. Strain sweeps from 0.1–100% strain at 2 Hz were also performed to determine the yielding properties of heterogeneous composites.

### 2.6 Degradation Evaluation

Hydrogel composites and bulk hydrogels with a 12 mm diameter and 4 mm thickness were fabricated using DLP. Samples were weighed and immersed in 2 mL of phosphate-buffered saline (PBS). At specific time points, samples were removed from PBS, washed with deionized water, and weighed after wiping off the excess water from the surface. The residual weight ratio was calculated (*W*_0_: the initial hydrogel weight; *W*_t_: the weight of the remaining, eq 1)


1 $$\mathrm{weight}\;\mathrm{residue}\;\left(\%\right)=W_{t}/W_{0}\times 100$$

### 2.7 DLP Printing

Different 2D shapes and 3D cross-sectional structures of human tissues were designed in Tinkercad and printed using a commercial DLP printer (LumenX+, CELLINK, Sweden) (100 μm layers, 80% power, 6 s exposure per layer, 2× base multiplier). Optical microscopy (ZEISS SteREO Discovery.V8) was performed to visualize the printed structures. To calculate the print fidelity, thickness measurements were taken along each line in the 2D grids and compared to the specified line width in the.stl file. To calculate printability, ImageJ was used to determine the perimeter (*L*) and area (*A*) of each square within the 2D grids. The printability was determined by applying the circularity equation to squares (eq 2) or hexagons (eq 3).

2 $$\mathrm{Pr}=L^{2}/16A$$

3 $$\mathrm{Pr}=\sqrt{3}L^{2}/24A$$

To determine photopolymerization depth, 1 mL of bioink was added to the ink reservoir and exposed to UV light for a duration equivalent to the exposure time used in printing. The thickness of the cross-linked layer was measured and assessed as the maximum depth that could be polymerized within the selected exposure time.

Organ analogs were printed using similar conditions. The 3D models were obtained from Thingiverse, used under the terms of the CC-BY Creative Commons Attribution 4.0 International License (https://creativecommons.org/licenses/by/4.0), from users “addamay123” (nose), “Cbonsig” (left ear) and “Misterxp” (little finger). For the scanning and reconstruction of the knee joint anatomical model, a scanning app (Qlone, EyeCue Vision Technologies Ltd.) was used. All models were modified using Blender 4.2 and Tinkercad to accommodate the size of the building platform and achieve optimal printing quality. Photos of printed constructs were taken with a Nikon D3500 camera, and original images were then processed using ImageJ.

### 2.8 Statistical Analysis

Quantitative data are presented as mean ± standard deviation (SD). Data were analyzed by GraphPad Prism 9. A paired *t* test was used to analyze the differences between the two groups of samples. One-way analysis of variance (ANOVA) was conducted to analyze the differences between three groups of samples. *p* < 0.05 was considered statistically significant.

## 3 Results and Discussion

### 3.1 Heterogeneous Hydrogel Composite Fabrication

Printable heterogeneous hydrogel composites were prepared by mixing precross-linked GelMA microgels with a GelMA-based bioink precursor containing LAP photoinitiator and tartrazine photoabsorber. As a biocompatible polymer, GelMA is widely used as a bioink for DLP 3D bioprinting.^77^ Based on the formulation of a commercial DLP bioink, a precursor was prepared containing 12.5 wt % GelMA, 15 mM LAP, and 1 mM tartrazine (CELLINK Bioprinting AB, Sweden). Microgels were prepared using a range of GelMA concentrations (7.5, 10, 12.5%). Using 12.5% GelMA in microgels ensured nanoscale uniformity between microgel and bulk phases, while the lower concentrations were prepared to explore how variation would affect composite properties. To prepare microgels, a high-throughput microfluidic droplet generator was fabricated. The device contains 100 parallelized step junctions and produces monodisperse microgel particles of ∼120 μm in diameter (Figure 1A) at a rate of 2 mL h^–1^. In step emulsification microfluidic devices, droplet size is independent of polymer concentration.^78^ This is evidenced by the consistency in microparticle diameter between formulations containing 7.5, 10, or 12.5% GelMA (Figure 1A). To form heterogeneous hydrogel composites, microgels were mixed with GelMA-based bioink precursor at different volume ratios before printing using DLP. Confocal imaging of printed composites shows that microgels remained stable during printing and could maintain their morphology (Figure 1B).

**Figure 1:**
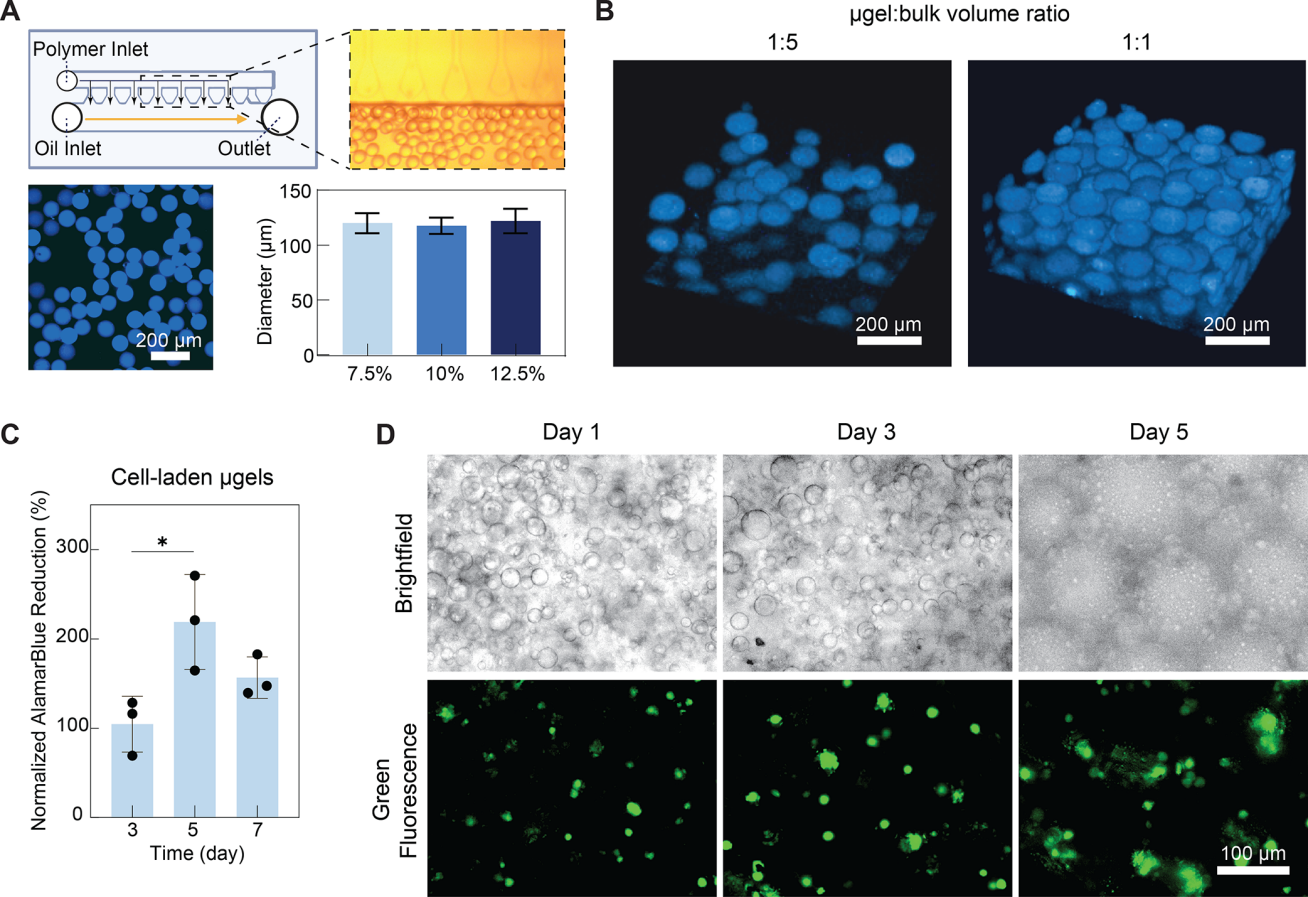
Fabricating heterogeneous hydrogel composites. (A) Preparation of microgels on a microfluidic chip under optical microscopy (top). Representative fluorescence image of microgels in suspension and average particle size of microgels with different GelMA concentrations (bottom). (B) Representative 3D reconstructions of confocal z-stacks of heterogeneous hydrogel composites with 1:5 (left) and 1:1 (right) μgel/bulk volume ratios. (C) AlamarBlue viability assay result for dermal fibroblast cells encapsulated in microgels after 3, 5, and 7 days of culture: normalized percentage alamarBlue reduction of cell-laden microgels. (D) Representative microscopic images of cell-laden composites after 1, 3, and 5 days of culture: brightfield (top) and green fluorescence (bottom). Statistical analysis performed using a one-way ANOVA, *p < 0.05.

To further explore the potential applications of microgels, we have utilized them to encapsulate fibroblasts. The cells exhibited good viability within 7 days following encapsulation (Figure 1C). Cell-laden composites were also fabricated using bioink composed of bulk and cell-laden microgels. The cells remained viable within the composites for 5 days after cross-linking (Figure 1D). The degradation of bulk and the swelling of microgels were observed. Further biological investigations, such as encapsulating extra bioactive factors in microgels in addition to cells, as well as functionalizing bulk, could enhance the resemblance of the system to the native tissues in terms of physicochemical properties and expand the scope of applications.

### 3.2 Nanoscale Optimization of Heterogeneous Hydrogel Composite

DLP printing occurs through a bottom-up technique in which layers of specified thickness are sequentially exposed to UV light at a certain intensity and time. During this process, several parameters can be tuned to control the mechanical performance of hydrogels. These include the concentration of polymer, photoinitiator, and photoabsorber, in addition to UV exposure energy. Modulation in these areas leads to nanoscale variations within the cross-linked polymer network, resulting in changes in macroscopic mechanical properties. Variations in cross-link density affect the ability of hydrogels to swell and absorb fluid, impacting mechanical stiffness, biodegradability, and therapeutic loading and release.^79–82^ We first explored the modulation of composite mechanical properties at the nanoscale by tuning cross-linking through polymer concentration in the embedded microgels.

#### 3.2.1 Polymer Concentration in Microgel

For a bioink to achieve proper functionality for DLP printing, the ink viscosity should allow for sufficient spreading across the building platform at printing temperatures. Optimal spreading is typically observed in an ink viscosity range of 10^0^ to 10^4^ mPa·s.^20,83^ Low temperatures increase the viscosity of GelMA-based bioinks, while excessive temperatures can lead to denaturation of polymer chains and negatively affect cell viability during bioprinting.^84–86^ Preliminary experiments were performed to assess the preprint fluidity of different composite ink formulations based on varying concentrations of GelMA within the microgels. With all other variables held constant, varying polymer concentrations of the microgels alone did not significantly affect the viscosity at printing temperatures. All experimental groups remained within a suitable viscosity above 30 °C (Figure 2B), which would prevent the physical gelation of GelMA during printing and provide cytocompatible conditions for any bioprinting applications.

**Figure 2:**
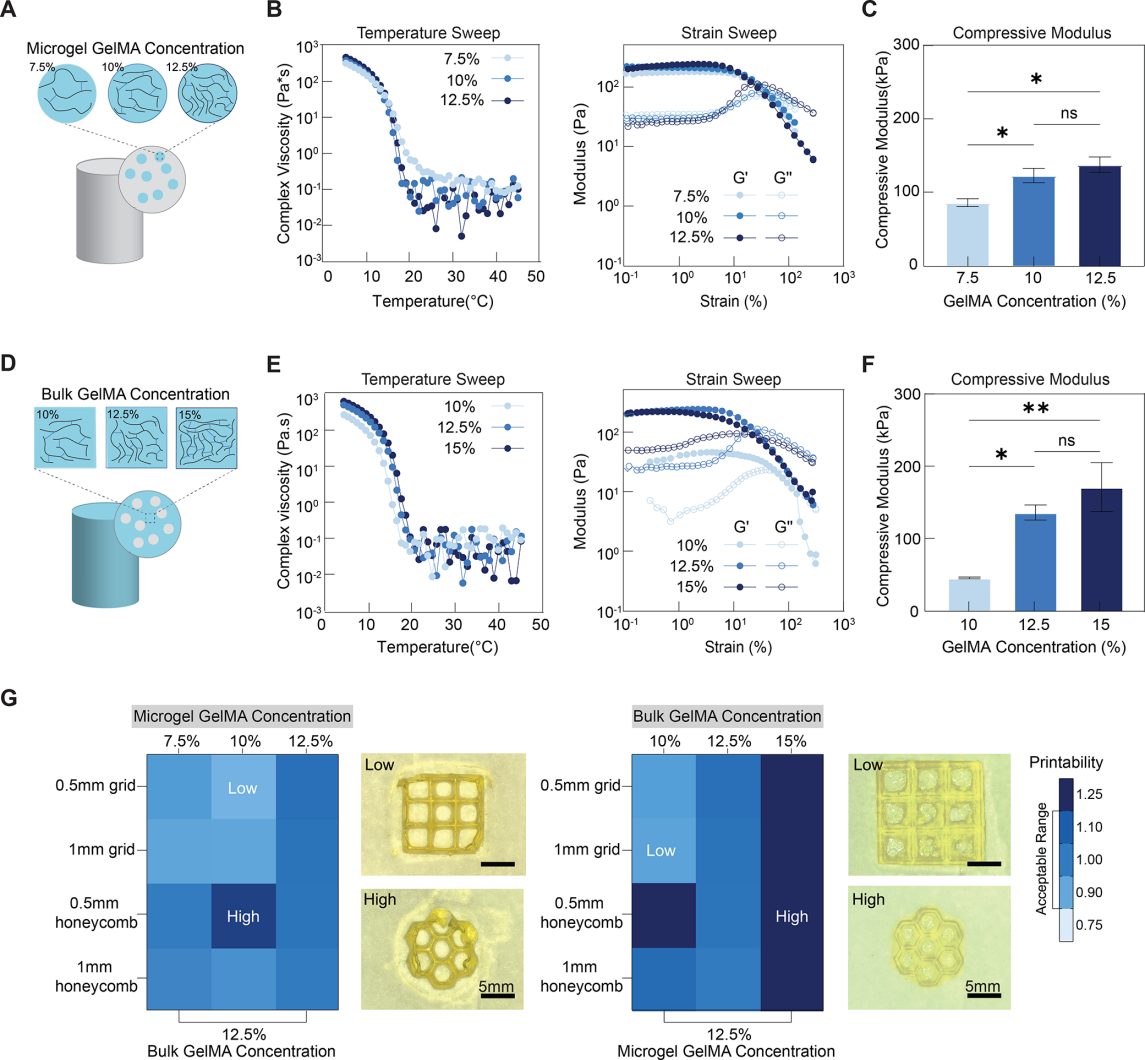
Influence of polymer concentration on heterogeneous hydrogel composite properties. (A) Schematic overview of influencing hydrogel composite properties by controlling microgel GelMA concentration. (B) Rheological characterization of heterogeneous hydrogel composites with varying microgel GelMA concentrations, including representative temperature sweeps for preprint composite bioinks (left, 4–45 °C) and strain sweeps for printed composites (right, 0.1–100%). (C) Compression test of heterogeneous hydrogel composites with varying microgel GelMA concentrations (*n* = 3): compressive moduli. (D) Schematic overview of influencing hydrogel composite properties by controlling bulk GelMA concentration. (E) Rheological characterization of heterogeneous hydrogel composites with varying bulk GelMA concentrations, including representative temperature sweeps for preprint composite bioinks (left, 4–45 °C), and strain sweeps for printed composites (right, 0.1–100%). (F) Compression test of heterogeneous hydrogel composites with varying bulk GelMA concentrations (*n* = 3): compressive moduli. (G) Representative printability heat maps for heterogeneous hydrogel composites with varying microgel GelMA concentrations (left) and bulk GelMA concentrations (right), each accompanied by images corresponding to the highest and lowest printability values. Images in (G) are reused in Figures S1B and S3B for fidelity and printability comparison. Statistical analysis performed using a one-way ANOVA, ns = no significance, *p < 0.05, **p < 0.01.

We then wanted to explore the effect of microgel composition on the rheological and mechanical properties of the printed composites. Studies have shown that embedding softer inclusions within a polymer network reduces the overall stiffness but improves yield strength and failure properties.^72^ Microgels with greater stiffness than the surrounding matrix result in an increased composite stiffness but reduced yield strength.^87^ Composite bioinks containing microgels with varying GelMA concentrations were applied to DLP 3D printing to fabricate heterogeneous hydrogel composites. Uniaxial compression to 60% strain was performed on printed constructs. In composites with a μgel/bulk volume ratio of 1:5, the addition of microgels reduced the compressive modulus of the resulting structure relative to the monolithic ink, regardless of microgel polymer concentration. Microgels acted as defects in the system, disrupting cross-linking throughout the network and weakening structural stability. This effect became more pronounced as the difference between polymer concentrations in the separate components increased. Adding microgels containing 12.5% GelMA to a bulk precursor with an identical amount reduced compressive modulus by approximately 25%. Meanwhile, the addition of microgels containing 7.5% GelMA to the same ink reduced the compressive modulus by 55% (Figure 2C). A potential explanation for this can be found in the microgel’s low stiffness, which prevents it from effectively resisting permanent deformation within the composite.

The results of the mechanical tests indicated a different effect on failure properties. Failure was observed at similar strains for each group, indicating the properties of the matrix dominated this aspect (Figure S1A). This is likely due to the low volume fraction of microgel inclusions. Unsurprisingly, a decrease in failure stress was observed as polymer concentration in microgels decreased. As the compressive moduli of composites were reduced, their ability to resist deformation due to applied stress was decreased. Rheological strain sweeps indicated that although the storage modulus of composites containing different microgels was initially similar, the yield strain increased as polymer concentration in the microgels decreased (Figure 2B).

In 3D bioprinting, fidelity and printability measurements are performed to determine the geometric accuracy of printed structures. Fidelity, measured by comparing printed line width to that of the specified geometry, describes the ability to reproduce computer-designed structures with high accuracy and precision. The ideal fidelity measurement is 1, while deviations indicate under or overcross-linking. Circularity equations can be applied to different shapes for a separate geometric accuracy assessment by defining a relationship between perimeter and area. The adherence of printed shapes to this relationship determines its printability. At both the micro- and macroscales, fidelity and printability together evaluate the overall printing performance.^88^

In the printing of 2D shapes, when GelMA concentration in the bulk was constant, changing microgel formulation had no significant effect on fidelity and printability. Moreover, at this bulk formulation, all experimental groups showed reduced line uniformity when printing shapes with a line width of 0.5 mm. As the microgel polymer concentration increased, this fluctuation in fidelity gradually diminished (Figures 2G and S1B). This phenomenon can be attributed to the presence of soft microgels, which introduce discontinuities within the matrix of the composite. Softer structures have shown limited compatibility with DLP due to their inability to support the weight of layers during printing. Further reduction of stiffness through the incorporation of microgels confirmed this relationship as printed structures became less accurate.

Overall, in comparison to monolithic bulk hydrogels with different GelMA concentrations (Figure S2A–C), the incorporation of microgels at lower volume ratios significantly impacted compressive moduli but did not show a similar effect on viscosity and geometric accuracy of DLP-printed shapes. This indicates the potential for designing composite bioinks with modular compressive moduli without affecting printability.

#### 3.2.2 Polymer Concentration in Bulk

In addition to varying polymer concentration in the microgels, we wanted to understand how making similar changes to the bulk precursor would affect composite properties. As polymer concentration increases, chain entanglements become more extensive, resulting in an increased cross-link density. Additionally, the entanglements may restrict the movement of the molecular chain and increase the shear modulus of the composite, thereby affecting the ink fluidity.^89^ This was assessed by performing a rheological temperature sweep on bioink precursors prepared using either 10, 12.5 or 15% GelMA. Similar to the previous results, changing bulk formulations within this range did not have a significant effect on preprint viscosity (Figure 2E).

To explore how bulk polymer concentration affected the stiffness of composites, different bulk formulations were mixed with 12.5% GelMA microgels at a μgel/bulk volume ratio of 1:5. Results of uniaxial compression to 60% strain indicated that, as expected, the concentration of GelMA in the ink directly affected its stiffness. After cross-linking, hydrogel composites with 12.5 and 15% GelMA in the bulk had comparable storage moduli and similar yield strains, while both properties were reduced with 10% GelMA (Figure 2E). Similarly, the use of 10% GelMA in the bulk led to significant reduction in compressive modulus compared to 12.5%, while an increase to 15% did not show a significant effect (Figure 2F). We then studied the effect of polymer concentration in the bulk on the printing performance of composite bioinks. Following the results of previous characterizations, the reduction of GelMA concentration in the bulk from 12.5 to 10% affected the printing of smaller structures, presumably due to insufficient mechanical stability of the composite bioink after cross-linking (Figures 2G and S3B). As a result, part of the printed construct was damaged during printing due to surface tension between the building platform and the bioink reservoir. On the other hand, while increasing bulk GelMA concentration to 15% did not result in any failed printing attempts, fidelity and printability were less ideal (Figures 2G and S3B). This is caused by increased viscosity leading to bioink aggregation in localized areas during printing, thereby increasing the risk of overcrosslinking.

#### 3.2.3 Photoabsorber Concentration

In DLP, photoabsorbers improve the resolution of printed structures by absorbing UV light energy and preventing cross-linking in areas outside of projected image layers. Therefore, photoabsorber concentration dramatically influences the degree of photopolymerization for ink. Tartrazine, a synthetic dye, is commonly used as a photoabsorber for DLP bioprinting due to its high absorbance of 405 nm light and its high cytocompatibility within a wide range of concentrations.^90,91^ In this experiment, both microgel and bulk GelMA concentrations were maintained at 12.5%, and three tartrazine concentrations (0.5, 1, and 2 mM) were explored for their effects on cross-linking kinetics, stiffness of printed composites, and fidelity and printability.

Rheological testing was performed to explore how varying photoabsorber concentrations would affect gelation kinetics of the composite bioinks. As expected, increasing the concentration of tartrazine prolonged the gelation time for the composite bioink, and correspondingly reduced the storage modulus of the cross-linked sample. At 0.5 mM tartrazine, the composite bioink exhibited a rapid increase in storage modulus within the first 10 s of UV exposure and reached a plateau after 25 s. Upon increasing tartrazine concentration to 1 mM, the onset of cross-linking was delayed and the rate of increase was slightly reduced. However, the exposure time required to achieve the maximum storage modulus was unaffected. When tartrazine concentration was increased to 2 mM, the gelation of the composite bioink was significantly delayed (Figure 3B). Moreover, the storage modulus was unable to reach similar levels after nearly 2 min of UV exposure. This is likely due to excess tartrazine absorbing a significant portion of UV energy, delaying the time at which a noticeable increase in storage modulus occurs. A similar trend was also observed when investigating how tartrazine concentration affected the maximum cure depth. Under the same UV intensity and exposure time, composite bioinks with 0.5 mM tartrazine resulted in cure depths exceeding 1000 μm. When using 2 mM tartrazine, a layer of only 250 μm was thoroughly cured after 1 min (Figure 3C).

**Figure 3:**
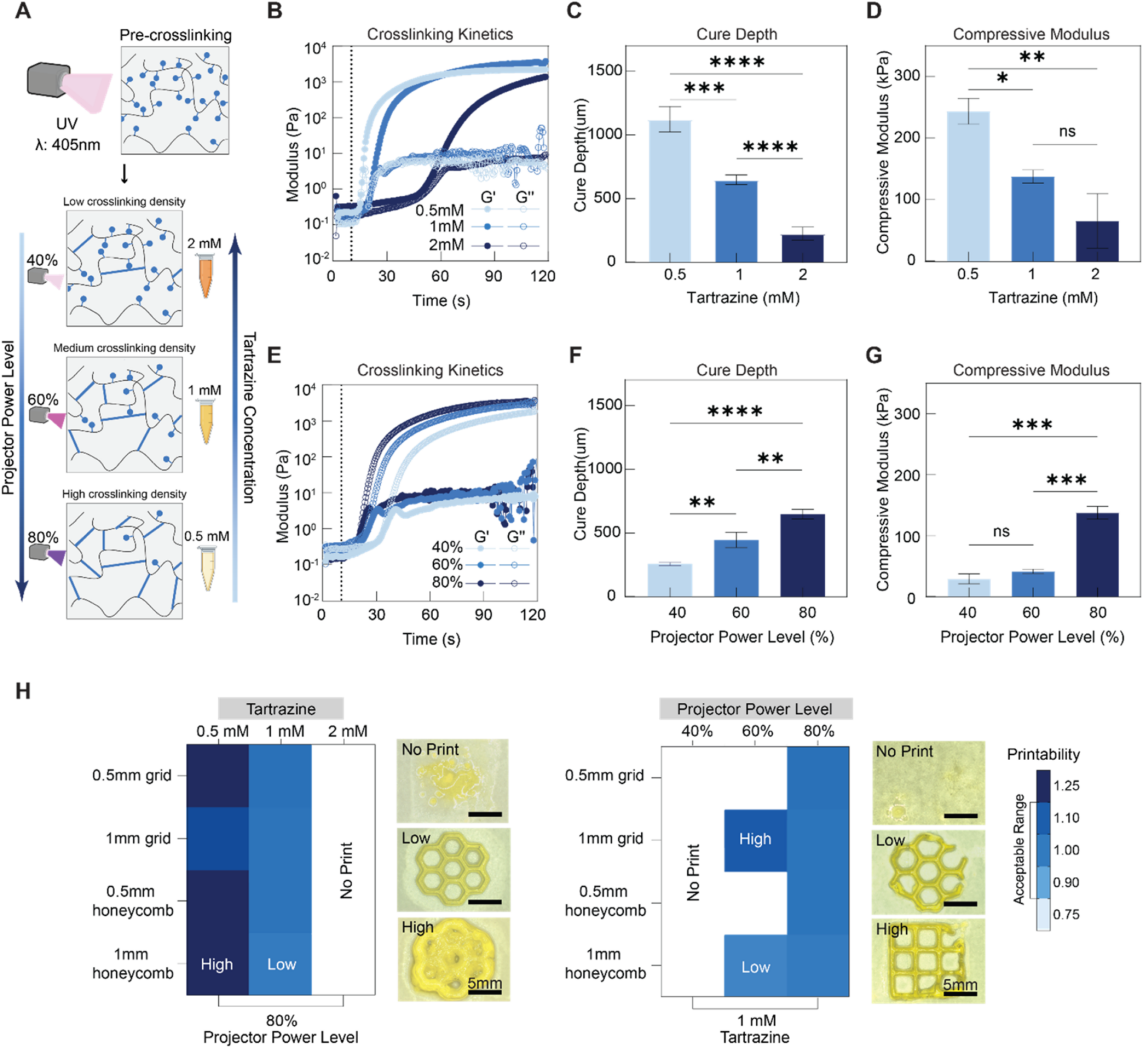
Influence of photopolymerization on heterogeneous hydrogel composite properties. (A) Schematic overview of influencing photopolymerization by controlling projector power level and tartrazine concentration. (B) Rheological characterization of heterogeneous hydrogel composites with varying tartrazine concentrations: representative results of UV-induced cross-linking using an 80% projector power level. (C) Assessment of the maximum photopolymerization depth under 0.5, 1, and 2 mM Tartrazine. (D) Compression test of heterogeneous hydrogel composites with varying tartrazine concentrations (*n* = 3): compressive moduli. (E) Rheological characterization of preprint hydrogel composite bioink: representative results of UV-induced cross-linking using 40, 60, and 80% projector power levels. (F) Assessment of the maximum photopolymerization depth under 40, 60 and 80% projector power levels. (G) Compression test of heterogeneous hydrogel composites printed using 40, 60 and 80% projector power levels (*n* = 3): compressive moduli. (H) Representative printability heat maps for heterogeneous hydrogel composites with varying tartrazine concentrations (left) and under varying projector power levels (right), each accompanied by images corresponding to the highest and lowest printability values, as well as failed printing attempts. All experiments were performed with 12.5% GelMA for both microgel and bulk concentrations. Images in (H) are reused in Figures S4B and S5B for fidelity and printability comparison. Statistical analysis performed using a one-way ANOVA, ns = no significance, *p < 0.05, **p < 0.01, ***p < 0.001, ****p < 0.0001.

Uniaxial compression tests to 60% strain were performed to evaluate the impact of tartrazine concentration on the mechanical strength of the printed structures. When using 0.5 mM tartrazine, the compressive modulus of the printed construct was around 250 kPa (Figure 3D). As the tartrazine concentration increased, a corresponding decline in modulus was observed, ultimately decreasing by 80% when tartrazine concentration was increased to 2 mM (Figure 3D). It is notable that with 2 mM tartrazine, the printed structures exhibited the same failure stress as those with 0.5 mM Tartrazine (Figure S4A). This may be attributed to the 2 mM tartrazine concentration causing a decrease in cross-link density, resulting in a softer printed construct. This reduction in stiffness with 2 mM tartrazine resulted in a composite able to undergo greater deformations at slower rates, displaying the same failure stress as stiffer constructs. This result was confirmed by the failure strain results, where an increase in tartrazine concentration resulted in a 20% increase in failure strain (Figure S4A).

Varying tartrazine concentrations led to significant effects on the fidelity and geometric accuracy of printed structures (Figures 3H, and S4B). Composites containing 1 mM Tartrazine achieved optimal fidelity and printability, forming constructs that highly resembled the CAD models. When the concentration was increased to 2 mM, excessive tartrazine absorbed the energy from UV exposure, preventing the initiation of photo-cross-linking. This composite bioink formulation was unable to print uniform shapes at either line thickness and instead formed randomly separated hydrogel fragments. With 0.5 mM tartrazine, shapes were printed with severely reduced fidelity and printability. While features of both honeycombs and grids were formed, significant overcrosslinking was observed during all printing attempts.

#### 3.2.4 UV Intensity

In DLP printing, increasing UV intensity transfers more energy to the same unit area, cross-linking constructs more rapidly. On the other hand, insufficient UV intensity can result in incomplete cross-linking, reducing the mechanical strength of the printed constructs and significantly compromising printing performance. An appropriate UV intensity can provide the proper energy to facilitate rapid cross-linking of layers to maintain sufficient mechanical properties without compromising fidelity and printability.

To explore the effects of UV intensity on the mechanical properties and printability of the composite bioinks, we initially investigated how variations would affect cross-linking kinetics, cure depth, and compressive modulus. The GelMA concentrations in both microgels and bulk were maintained at 12.5%, with tartrazine held at a constant 1 mM. Initially, three different UV intensities were selected based on the power levels of a LumenX+ bioprinter. Specifically, 40, 60, and 80% power correspond to 4, 7.8, and 11 mW/cm^2^, respectively. Rheological assessments indicated that at all UV intensities, a significant rise in storage modulus occurred within 30 s after UV exposure. However, 40% power did not supply enough energy for the composite to reach a storage modulus plateau within 2 min (Figure 3E). Typically, DLP printing is performed using exposure times on the range of 0.5 to 30 s per layer. Within that range, this low amount of UV energy would result in incomplete cross-linking and a significant reduction in stiffness. The cure depth experiment further confirmed this limitation. After 1 min of UV at 80% power, a layer of approximately 600 μm was cured, while 40% power only cured around 250 μm (Figure 3F).

Mechanical testing through uniaxial compression to 60% strain determined that a reduction in UV exposure affected the moduli of printed composites. The use of 40% and 60% power led to structures with moduli that were significantly reduced compared to constructs printed at 80%. Within the limited exposure time, the energy delivered at these power levels was insufficient to induce adequate cross-linking, resulting in a less stable network. Similar to previous results, softer constructs were able to withstand the same compressive force as stiffer composites by undergoing greater deformation. Failure strain was inversely proportional to UV exposure, as the constructs with a reduced stiffness were able to undergo slightly increased levels of deformation (Figure S5A). No direct relationship between UV exposure and failure stress was observed.

Printing of 2D shapes was performed to determine the optimal level of UV exposure for geometric accuracy. (Figures 3H, and S5B) Using 40% power did not result in successful printing of any attempted shape. This is due to insufficient cross-linking in printed layers, hindering their ability to support the weight of the following layers as the structure was built, resulting in print failure.^47^ The 60% power level failed to print any shapes with a 0.5 mm line width but was able to print shapes with a 1 mm line width to an extent, although the fidelity and printability were not ideal. In contrast, 80% power resulted in successful prints of all four shapes with good fidelity and printability. These results allow for the optimization of cross-link density through UV exposure rather than varying material-related parameters.

### 3.5 Engineering Microscale Heterogeneity in Printed Composites

#### 3.5.1 Volume Fraction

To study the effect of microgel volume fraction on the heterogeneous hydrogel composite system, we prepared composite bioinks with three different μgel/bulk volume ratios (1:5, 1:1, 3:1). This ratio directly affects the microstructure of composites, as interactions between these two components become more or less pronounced. At a μgel/bulk volume ratio of 1:5, the bulk dominated the heterogeneous hydrogel composite, with the microgels acting as defects embedded within the continuous phase and influencing the bulk’s behavior. At 3:1, the microgels occupied the majority of the volume in the hydrogel composite system. In this case, the properties of the composite bioink resembled those of a granular hydrogel with interstitial fluid filling the interparticle voids.

The 1:5 volume ratio was intended to represent the effect of a small amount of microgel inclusions on the composite properties. At 1:1 volume ratio, the microgel and bulk occupy the same volume in the system and were expected to have equal impacts on the properties of the hydrogel composite. A 3:1 volume ratio was prepared to investigate the properties of the composite bioink when microgel serves as the primary component. At this ratio, microgels comprised 75% of the total volume, which is the maximum packing density achievable for spherical particles.^41^ Beyond this limit, softer gel particles may undergo deformation during packing. SEM was utilized to examine the composites’ microstructure and the interfacial architecture between microgels and bulk (Figure S6). At a 1:5 volume ratio, a smoother, more continuous bulk matrix was observed, with microgels throughout and pores that formed during lyophilization. As the microgel content increased, the continuity of the bulk became disrupted. At a 3:1 volume ratio, the composite was mainly composed of microgels, with the bulk appearing as a thin, mesh-like coating over the surface of packed microgels.

Temperature sweeps showed that at 1:5 volume ratio, the bulk had a more dominant influence on its properties. The fluidity of the composite remained unchanged compared with the bioink without any microgel. A similar effect was observed at the 1:1 volume ratio, which showed comparable viscosities. In the composite bioink with a 3:1 volume ratio, the microgels dominated the viscous properties. Even after the temperature exceeded 40 °C, the viscosity of the 3:1 composite was not within a printable range (Figure S7A).

The gelation tests demonstrated that as microgel volume fraction increased, the time required for the storage modulus to reach its maximum was prolonged. Compared with the composite bioinks with 1:5 and 1:1 volume ratios, those with a 3:1 ratio had a slightly higher storage modulus prior to UV irradiation, which is aligned with the results of the temperature sweeps. Moreover, the composite bioink with a 3:1 volume ratio required more exposure time before an increase in modulus was observed, which we hypothesized was caused by microgels preventing the formation of a uniform network and slowing the gelation kinetics of the composite. The 3:1 volume ratio also resulted in the smallest difference in modulus before and after UV exposure. An opposite trend was observed in the composite bioink with a 1:5 volume ratio. This bioink showed a lower viscosity before UV exposure and reached a storage modulus of 100 Pa within 20 s of exposure, compared to more than 45 s at a 3:1 volume ratio (Figure 4C).

**Figure 4:**
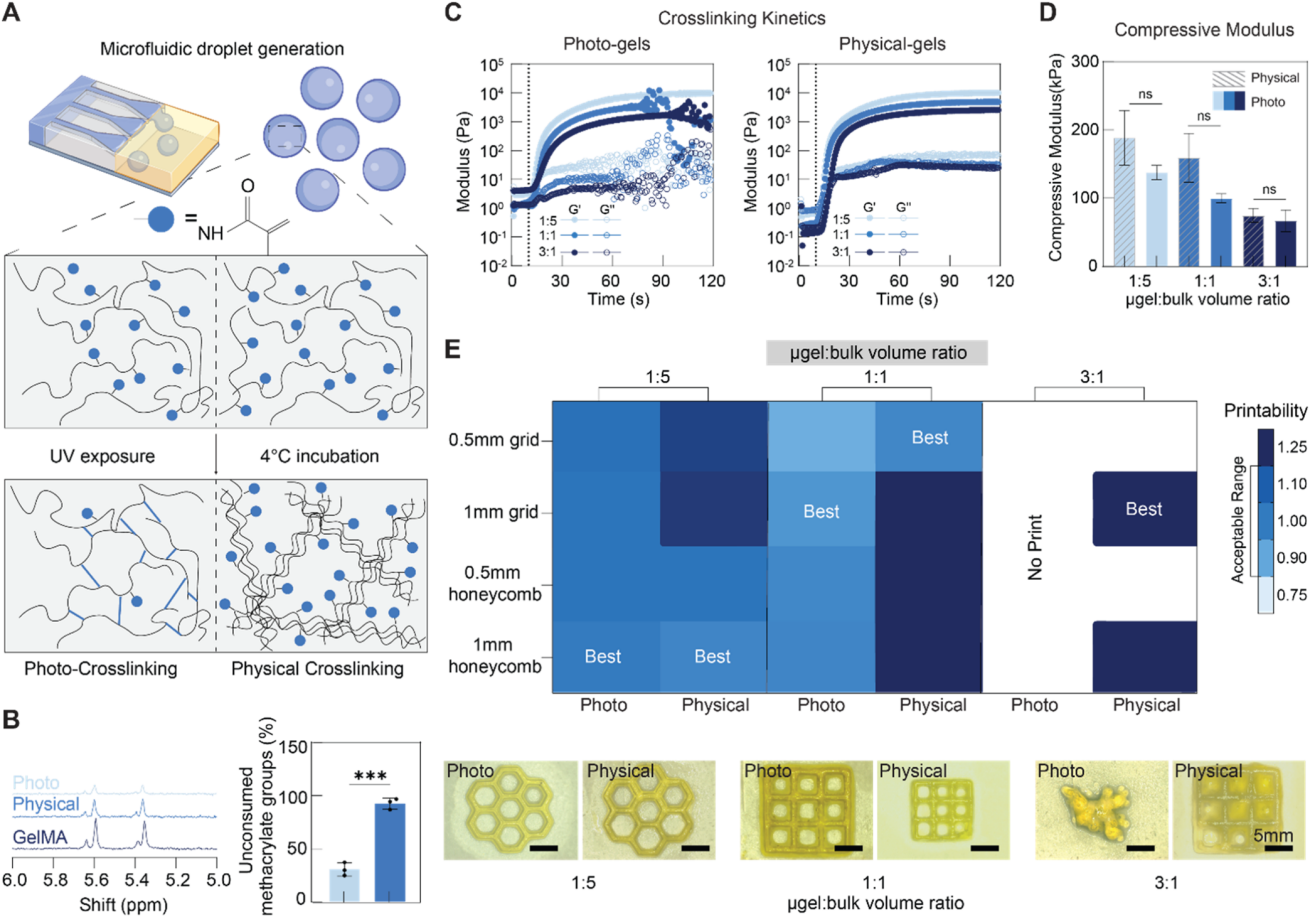
Influence of microgel-bulk interactions on heterogeneous hydrogel composite properties. (A) Schematic overview of microfluidic droplet generation and cross-linking mechanism of microgels via photo-cross-linking and physical cross-linking. (B) ^1^HNMR spectra of un-cross-linked GelMA and microgels both photo-cross-linked and physically cross-linked (left), and quantification of unconsumed methacrylate groups in photo-cross-linked and physically cross-linked microgels (right). (C) Rheological characterization of preprint hydrogel composite bioink containing photo-cross-linked microgels (left) and physically cross-linked microgels (right): representative results of UV-induced cross-linking using 80% projector power level. (D) Compression test of heterogeneous hydrogel composites containing photo-cross-linked and physically cross-linked microgels (*n* = 3): compressive moduli. (E) Representative printability heat map for heterogeneous hydrogel composites with varying microgel cross-link types and microgel to bulk volume ratios (top), and images corresponding to printability values closest to 1 are considered to exhibit the best printing performance. (bottom). All experiments were performed with 12.5% GelMA for both microgel and bulk concentrations. Images in (E) are reused in Figures S7C and S9B for fidelity and printability comparison. Statistical analysis performed using a one-way ANOVA, ns = no significance, ***p < 0.001.

The compressive moduli of printed constructs were negatively correlated with the microgel volume fraction. Uniaxial compression tests to 60% strain determined that as the volume fraction of microgels increased, the compressive modulus of printed composites decreased significantly. Volume ratios of 1:5 μgel/bulk produced structures with an average modulus of approximately 140 kPa, while 3:1 volume ratios led to softer structures of ∼66 kPa (Figure 4D). Failure stresses of the composite structures followed a similar trend. However, the failure strain was independent of the volume ratio, indicating that microgels did not affect the deformability of printed constructs, but influenced its energy absorption capacity under the same levels of deformation (Figure S7B). The granular nature of the packed microgels in printed constructs with a 3:1 volume ratio resulted in lower failure stress. In contrast, composite bioinks with lower microgel content could absorb more energy as well as withstand and distribute greater forces. Additionally, the higher microgel volume fraction affected the degradation rate of the composites. Composites with a 1:1 volume ratio degraded within 4 days, whereas those with a 1:5 ratio showed improved stability comparable to monolithic bulk hydrogels (Figure S8).

Printability assessments supported these findings, showing that 1:5 and 1:1 μgel/bulk volume ratios allowed the fabrication of structures with reduced geometrical accuracy. On the other hand, 3:1 μgel/bulk volume ratio significantly reduced the preprint fluidity of the composite as well as its capability to produce specified printing features. (Figures 4E, and S7C) Fidelity and printability of 2D shapes were optimal at the 1:5 ratio, however, acceptable print quality was obtained when printing with a 1:1 ratio. Upon increasing the microgel fraction to 75%, none of the attempted prints was successful.

#### 3.5.2 Physically Cross-Linked Microgels

Up to this point, the microgels used to fabricate the heterogeneous composites were formed using UV-triggered covalent cross-linking. This cross-linking process reduces the number of reactive methacrylate groups in the microgels, limiting their ability to interact and integrate with the bulk during the printing of composites. To address this and attempt to increase the interaction of microgels with the surrounding matrix, microgels were physically cross-linked through incubation at 4 °C without prior UV exposure and combined with bioink precursors to prepare composites (Figure 4A).^92^ When physically cross-linked microgels were incorporated into the bulk, more functional groups were available to react with the matrix under UV light exposure (Figure 4A,4B), strengthening μgel-bulk interactions. Additionally, the mechanical strength of the printed constructs was enhanced due to the stronger bonding between microgel and bulk. Uniaxial compression to 60% strain confirmed that at similar volume ratios, printed constructs containing physically cross-linked microgels had higher compressive modulus and failure stresses than those containing photo-cross-linked microgels.

Rheological assessments of gelation kinetics showed that a similar trend was observed with physically cross-linked microgels as compared to those that were photo-cross-linked. As microgel volume increased, a longer exposure time was required for an increase in storage modulus to be observed. With physically cross-linked microgels, an increase in maximum storage modulus was achieved when volume fraction was kept constant (Figure 4C). Assessments of compressive modulus (Figure 4D) and failure properties (Figure S9A) showed that the volume fraction-dependent reduction in stiffness observed previously was consistent. However, the increase in available methacrylate groups led to an increase in the average compressive modulus of composites at all volume ratios. This is likely due to increased interactions with the surrounding bulk.

The addition of physically cross-linked microgels slightly improved the print resolution of composites compared to photo-cross-linked gels (Figure 4E). However, fidelity and printability were both still negatively impacted by increases in microgel content. At 1:5 and 1:1 μgel/bulk volume ratios, composite bioinks with physically cross-linked microgels showed similar performance to those containing photo-cross-linked microgels. However, the printability and fidelity were improved at the 3:1 volume ratio, indicating stronger interactions between embedded microgels and the surrounding bulk (Figures 4E, and S7C, S9B).

### 3.6 Engineering Macroscale Heterogeneity through Printing

Apart from the variables explored, it is possible to further modify composite properties through UV exposure time. However, increasing cross-link density through increasing exposure time can significantly increase the overall printing time. When printing at elevated temperatures, this prolonged printing time may lead to water loss from the bioink, resulting in brittle structures or failed prints. To circumvent these issues, major changes to the printer design and peripheral equipment are often required, which can be costly and hinder the reproducibility of results. Therefore, in this study, the exposure time used for printing was based on standard printing conditions for the commercial bioink on which the formulation was based.

Additionally, the concentration and type of photoinitiator can be varied to modify printable hydrogel systems. LAP is usually used in DLP 3D bioprinting for its satisfactory cell compatibility and high reactivity to 405 nm UV light.^93,94^ Optimal LAP concentrations are typically within the range of 5 to 40 mM^48,95,96^ to support rapid gelation and sufficient mechanical stiffness, while concentrations ranging from 5 to 15 mM^93,97,98^ are commonly used for printing with encapsulated cells. In this study, 15 mM LAP was chosen for the bioink formulation.

Through exploring how different parameters such as polymer or photoabsorber concentration, volume fraction of composite components, or UV exposure affect composite properties, an optimal ink formulation was determined. By using a composite with a 1:5 μgel:bulk volume ratio, containing similar polymer concentrations in both components (12.5% GelMA), a tartrazine concentration of 1 mM, and printing with 11 mW/cm^2^, structures were fabricated with ideal geometric accuracy. Therefore, these parameters and formulations were utilized to print various 3D biological structures with high resolution.

3D cross-sectional structures of human tissues were first printed (Figure 5A), demonstrating good shape fidelity. However, achieving high resolution in small surface features, especially those with gaps and cavities, proved to be challenging (Figure 5A). Subsequently, different organ models were printed to further demonstrate the capability of the hydrogel composite bioink in printing intricate 3D structures. Compared to 2D shapes and cross-sectional structures in which each layer is identical, individual layers of organ models are unique, resulting in a structure that varies in all three dimensions. Successful reproduction of these details requires optimized mechanical properties combined with appropriate printing parameters to ensure the integrity of printed constructs and prevent structural failure, which is often caused by the insufficient strength of GelMA.^47^ The results show that the structures can be printed with good integrity and high similarity to the 3D model. Intricate details such as nostrils and fingernails can be observed from the printed structures (Figure 5B). Across all types of 3D printing, modeling often requires extensive training in modeling software. To facilitate faster design processes and achieve personalized 3D printing applications, 3D scanning applications can be used to reconstruct and export 3D models of real-world objects with minimal effort.^43^ A knee joint model was scanned, and a .stl file was created and exported through the app for DLP printing (Figure 5C). In addition, we explored the feasibility of multimaterial printing, using 2 or 3 inks, constructs with morphological and mechanical heterogeneity can be fabricated (Figure 5D,E), with different regions displaying varying colors and compressive moduli.

**Figure 5:**
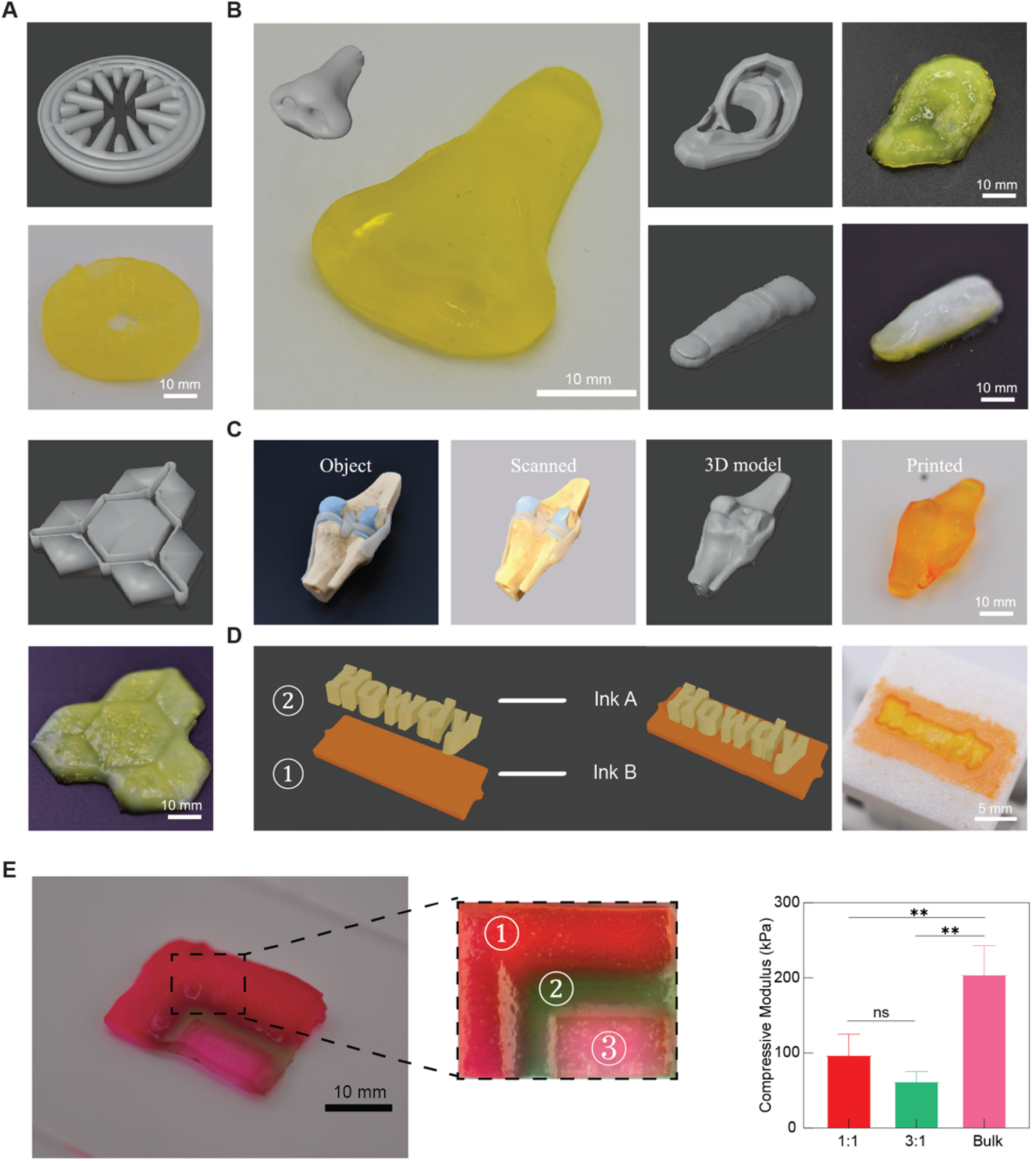
Constructs printed using the heterogeneous hydrogel composite bioinks. (A) 3D models and printed constructs of cross-sectional structure of human tissues: small intestine (top) and hepatic lobules (bottom). (B) 3D models and printed constructs of organ-analogues: nose (left), left ear (top right), and little finger (bottom right). (C) 3D model obtained from the scanning app and printed structure of a knee joint anatomical model. (D) Schematic overview of dual-material printing using 2 bioinks (left), and the printed structure (right). (E) Structure printed using 3 different bioinks (left), interfaces between different bioinks (center), and compressive modulus of regions corresponding to each bioink (right). Statistical analysis performed using a one-way ANOVA, ns = no significance, **p < 0.01.

## 4 Conclusions

A dual-component, DLP printable hydrogel composite platform with multiscale heterogeneity was developed. The GelMA concentration of the microgels and bulk phase was varied to study the effect of nanoscale cross-link density on the mechanical and rheological properties, as well as printability and fidelity. The microscale microgel/bulk volume ratio and different cross-linking methods influenced the gelation kinetics, mechanical properties, and printing performance of the heterogeneous hydrogel composite. Lastly, a hydrogel composite bioink formulation with optimal printing performance was utilized for the DLP printing of biological structures, which showed significant similarity to the native tissue architecture. Multimaterial printing was also explored as a strategy to fabricate constructs with macroscale morphological and mechanical heterogeneity. The results suggest that this unique platform can be leveraged to develop bioactive tissue models for various regenerative medicines. This work demonstrated the impact of multiscale heterogeneity in printing and explored multimaterial printing. These findings will benefit the design of hydrogel composites with tunable properties for 3D bioprinting and other biomedical applications.

## Supplementary Material



## Data Availability

All the raw data is available over Zenodo: 10.5281/zenodo.15376346.

## Notes

Heterogeneous hydrogel composite (PDF)
