# Supplementary material for: Multiscale Engineered Heterogeneous Hydrogel Composites for Digital Light Processing 3D Printing
Source: ACS Appl Mater Interfaces. 2025 Sep 8;17(37):51605–19. doi: 10.1021/acsami.5c09635 (PMC12447404; doi:10.1021/acsami.5c09635)
Supplement: Supplementary file 1 [file am5c09635_si_001.pdf]

Supporting Information

Multiscale Engineered Heterogeneous Hydrogel Composites for Digital Light Processing 3D Printing

Yuang Zhang[1‡], Ryan Davis, Jr.[2‡], Saptarshi Biswas[2], Sarah E. Miller[2], Syed Raza Ur Rehman[2], Gene T. Felix[2], Akhilesh K. Gaharwar[1,2,3,4]*

[1] Department of Materials Science and Engineering, College of Engineering, Texas A&M University, College Station, TX, 77843, USA.

[2] Department of Biomedical Engineering, College of Engineering, Texas A&M University, College Station, TX, 77843, USA.

[3] Interdisciplinary Program in Genetics, Texas A&M University, College Station, TX, 77843, USA.

[4] Center for Remote Health Technologies and Systems, Texas A&M University, College Station, TX, 77843, USA

[‡] These authors contributed equally to this work.

Corresponding Author: Akhilesh K. Gaharwar - Email: gaharwar@tamu.edu

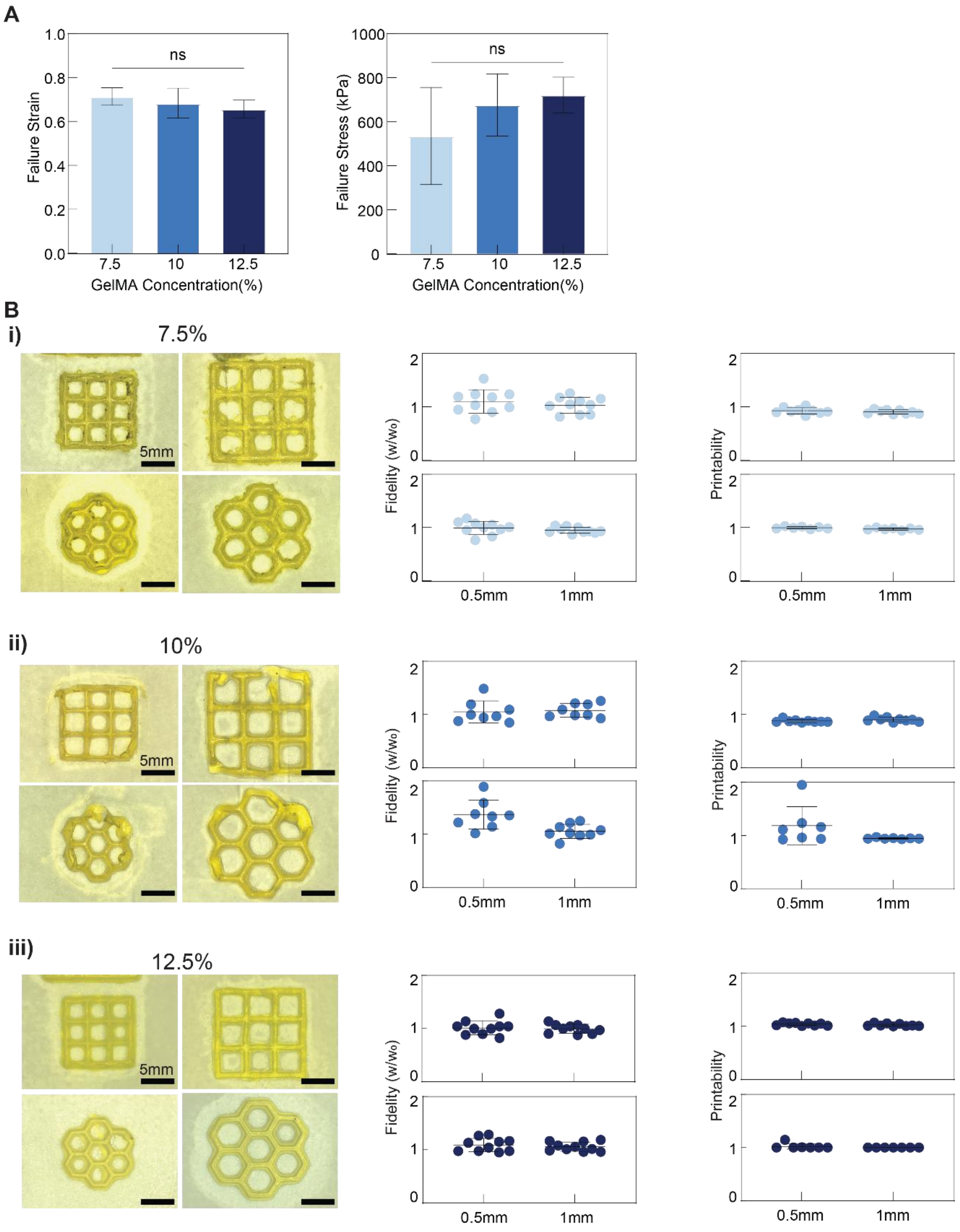

Figure S1. A Compression test of heterogeneous hydrogel composites with 12.5% bulk GelMA concentration and varying microgel GelMA concentrations (n=3): failure strain (left) and failure

stress (right). **B** Printing of composite bioink with 12.5% bulk GelMA concentration and varying microgel GelMA concentrations (**i)** 7.5%, **ii)** 10%, **iii)** 12.5%), including images of printed structures (left), quantification of fidelity (center), and quantification of printability (right). Images from **Fig. 2G** are reused for fidelity and printability comparison. Statistical analysis performed using a one-way ANOVA, ns = no significance.

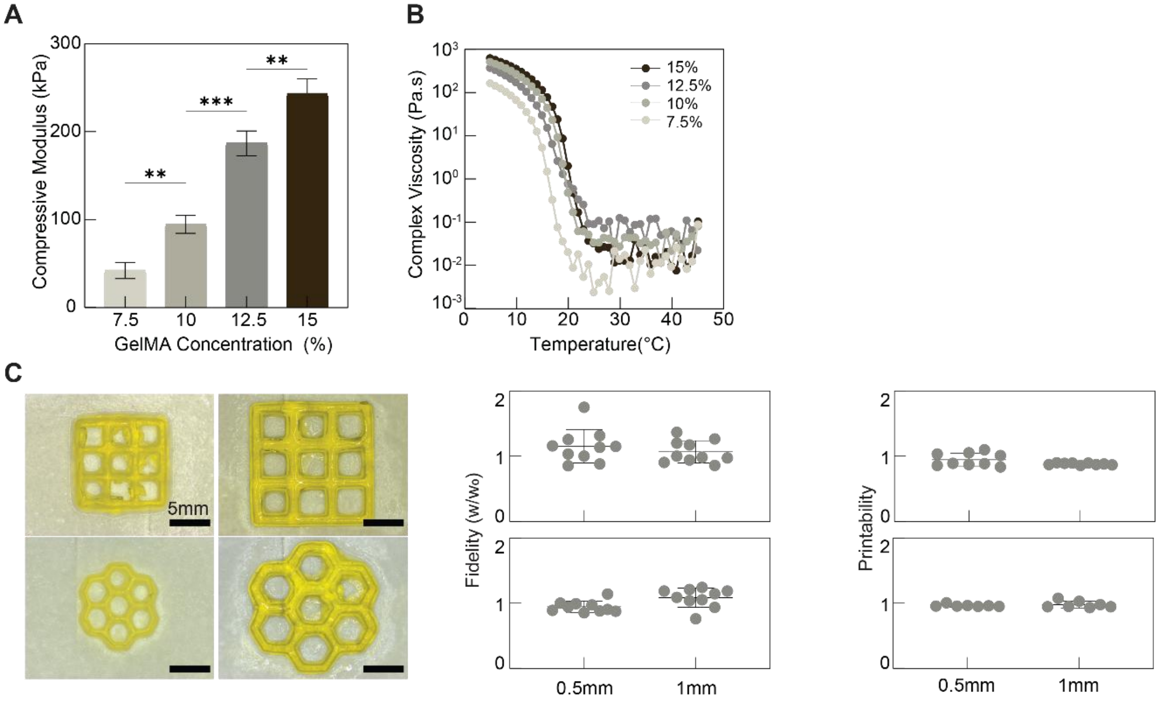

Figure S2. A Compression test of bulk hydrogels with varying GelMA concentration (n=3): compressive modulus. **B** Rheological characterization of bulk hydrogels with varying GelMA concentrations: temperature sweeps for pre-print bulk bioinks (4-45°C). **C** Printing of bulk bioink with 12.5% GelMA concentration, including images of printed structures (left), quantification of fidelity (center), and quantification of printability (right). Statistical analysis performed using a one-way ANOVA, $**p < 0.01$, $***p < 0.001$.

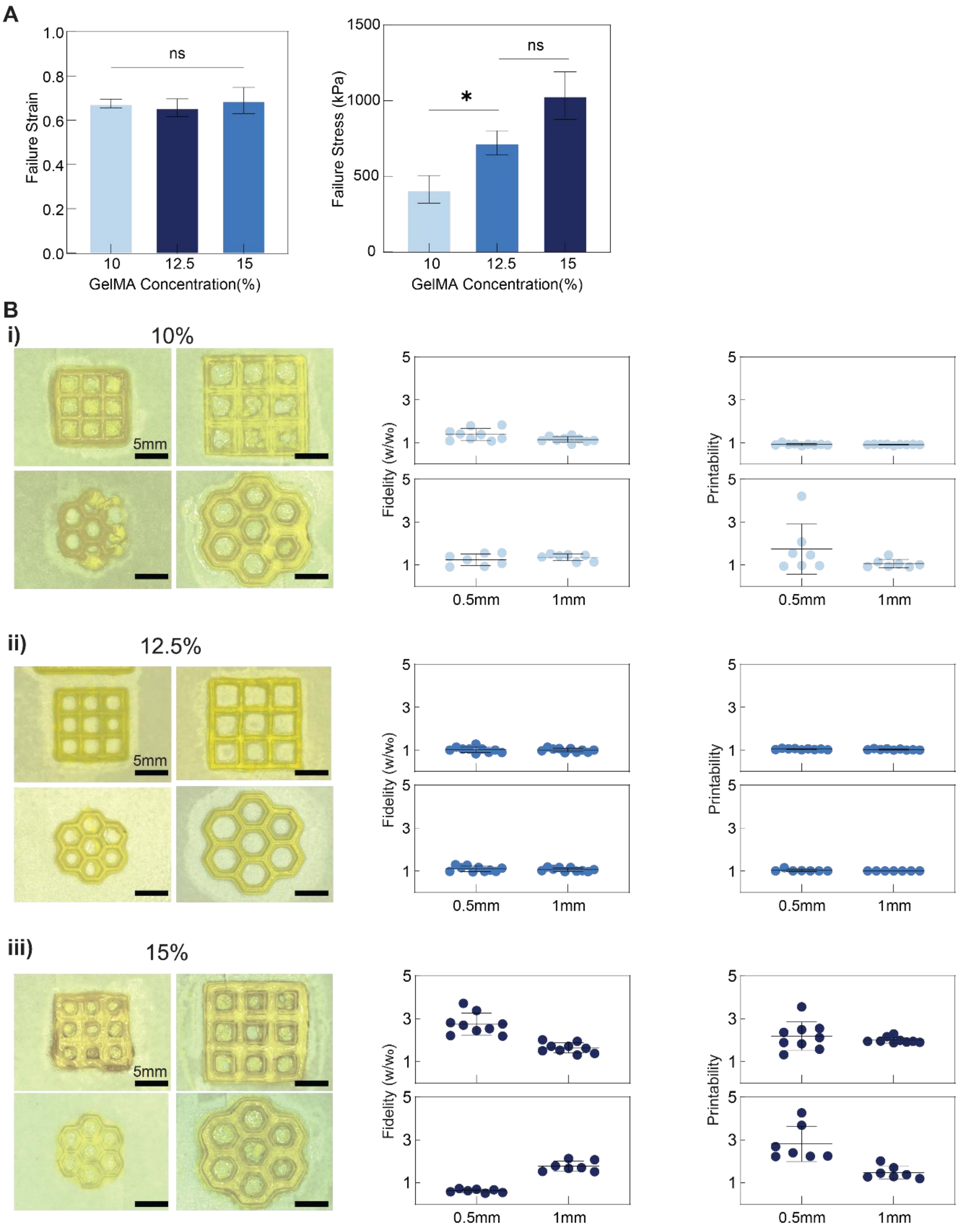

Figure S3. A Compression test of heterogeneous hydrogel composites with 12.5% microgel GelMA concentration and varying bulk GelMA concentrations (n=3): failure strain (left) and

failure stress (right). **B** Printing of composite bioink with 12.5% microgel GelMA concentration varying bulk GelMA concentrations (**i)** 10%, **ii)** 12.5%, **iii)** 15%), including images of printed structures (left), quantification of fidelity (center), and quantification of printability (right). Images from **Fig. 2G** are reused for fidelity and printability comparison. Statistical analysis performed using a one-way ANOVA, ns = no significance, *$p < 0.05$.

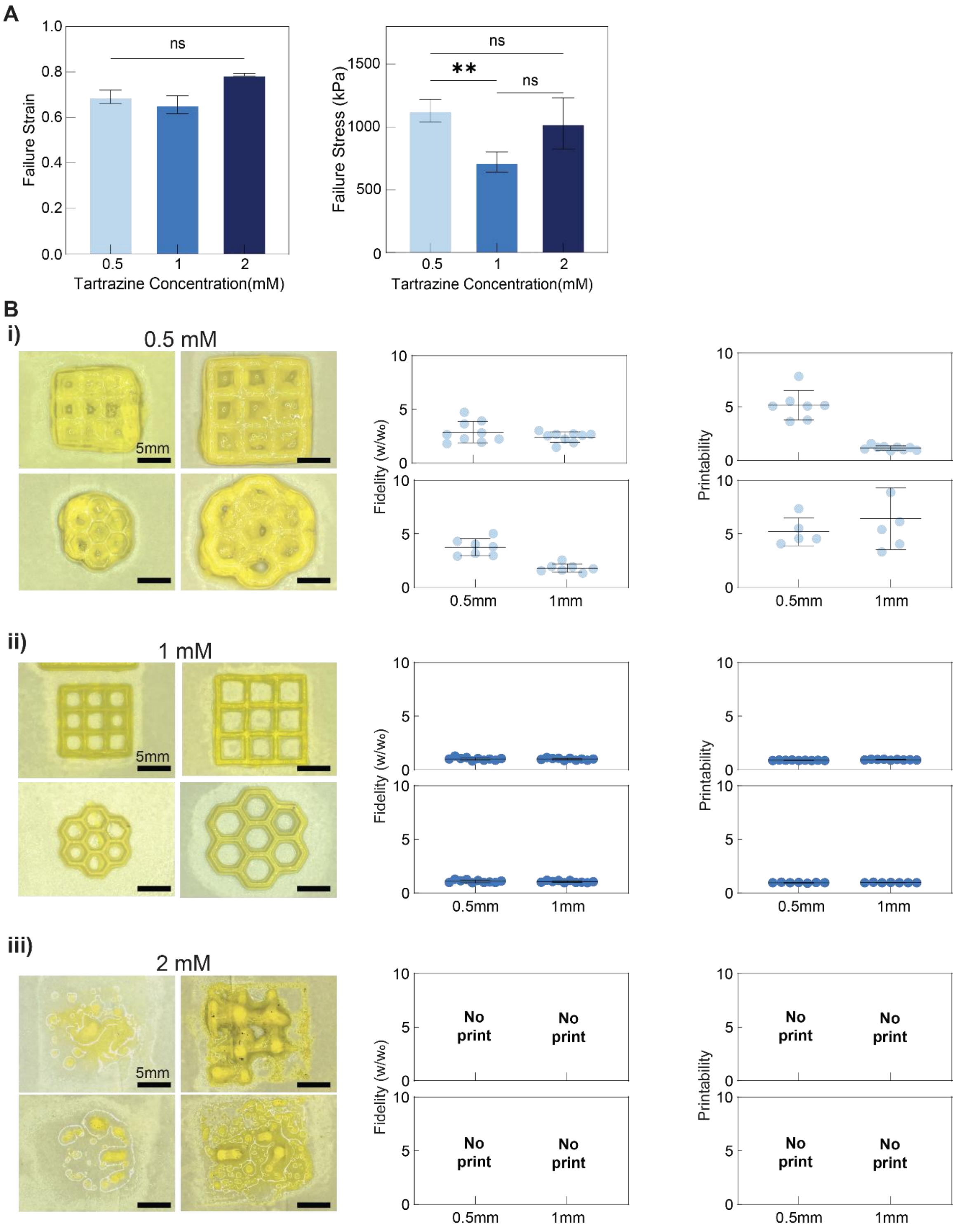

Figure S4. A Compression test of heterogeneous hydrogel composites with varying Tartrazine concentrations (n=3): failure strain (left) and failure stress (right). **B** Printing of composite bioink

with varying Tartrazine concentrations (**i)** 0.5 mM, **ii)** 1 mM, **iii)** 2 mM), including images of printed structures (left), quantification of fidelity (center), and quantification of printability (right). All experiments were performed with 12.5% GelMA for both microgel and bulk concentrations. Images from **Fig. 3H** are reused for fidelity and printability comparison. Statistical analysis performed using a one-way ANOVA, ns = no significance, $^{**}p < 0.01$.

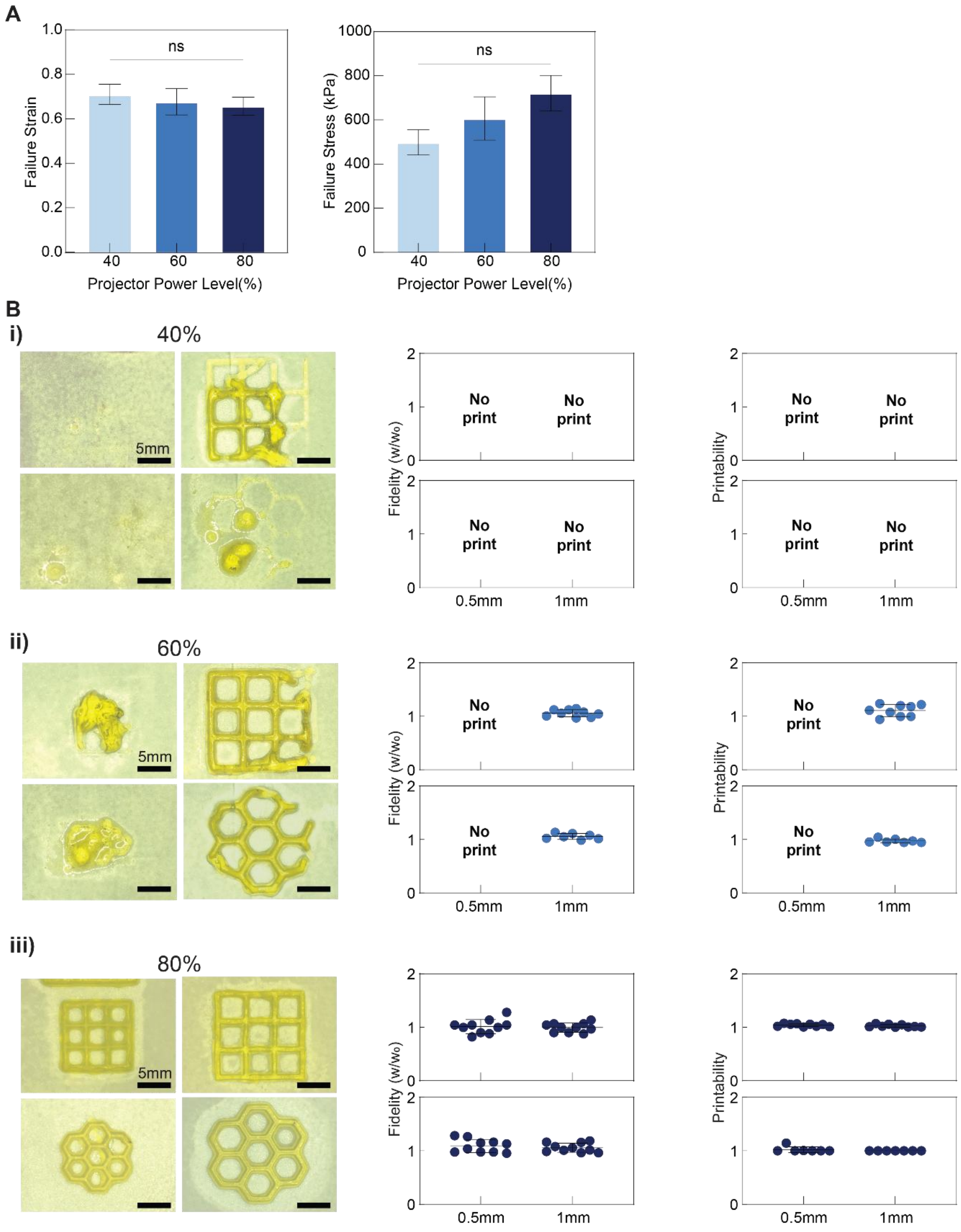

Figure S5. A Compression test of heterogeneous hydrogel composites printed with varying projector power levels (n=3): failure strain (left) and failure stress (right). **B** Printing of

composite bioink using varying projector power levels (**i)** 40%, **ii)** 60%, **iii)** 80%), including images of printed structures (left), quantification of fidelity (center), and quantification of printability (right). All experiments were performed with 12.5% GelMA for both microgel and bulk concentrations. Images from **Fig. 3H** are reused for fidelity and printability comparison. Statistical analysis performed using a one-way ANOVA, ns = no significance.

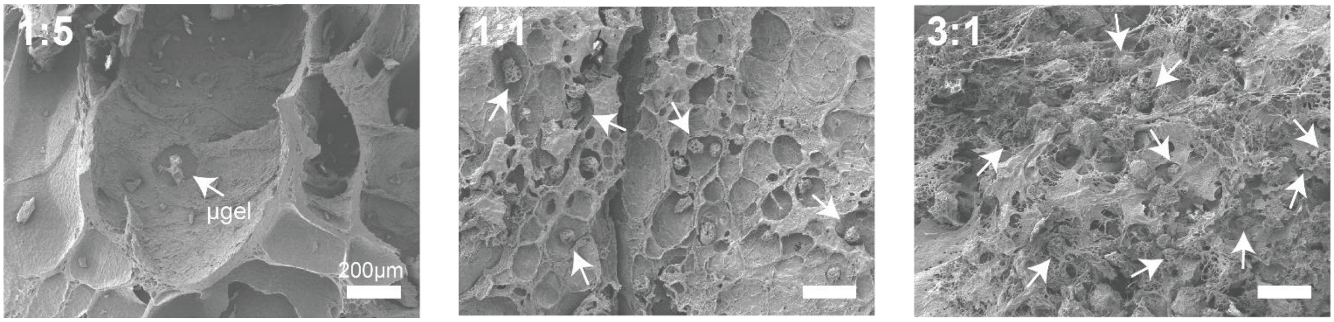

Figure S6. Scanning electron microscopy images of heterogeneous hydrogel composites with 1:5 (left), 1:1 (center), and 3:1 (right) photocrosslinked microgel to bulk volume ratios.

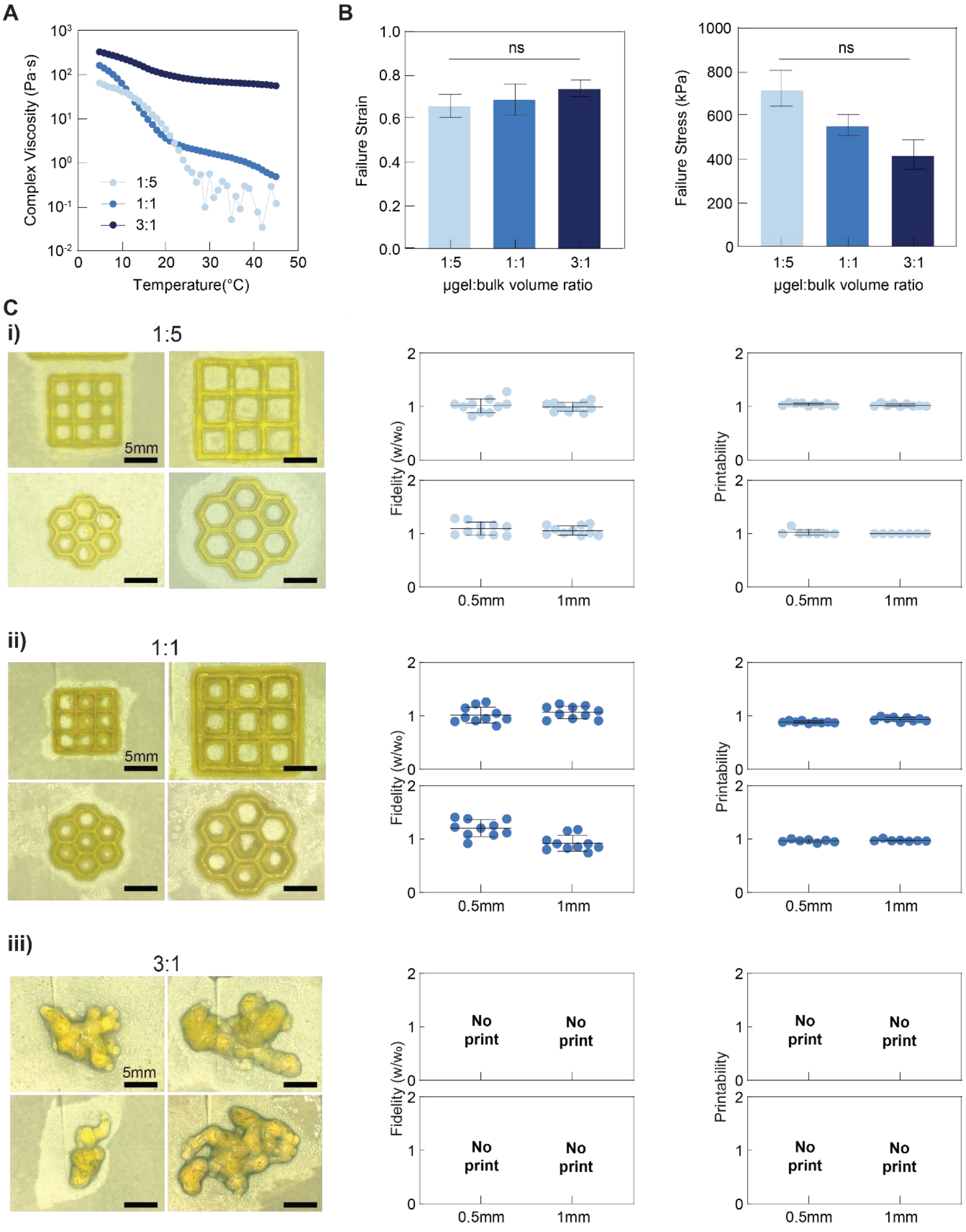

Figure S7. A Rheological characterization of heterogeneous hydrogel composites with varying photocrosslinked microgel to bulk volume ratios: temperature sweeps for pre-print composite

bioinks (4-45°C). **B** Compression test of heterogeneous hydrogel composites printed with varying photocrosslinked microgel to bulk volume ratios (n=3): failure strain (left) and failure stress (right). **C**: Printing of composite bioink with varying photocrosslinked microgel to bulk volume ratios (**i)** 1:5, **ii)** 1:1, **iii)** 3:1), including images of printed structures (left), quantification of fidelity (center), and quantification of printability (right). All experiments were performed with 12.5% GelMA for both microgel and bulk concentrations. Images from **Fig. 4E** are reused for fidelity and printability comparison. Statistical analysis performed using a one-way ANOVA, ns = no significance.

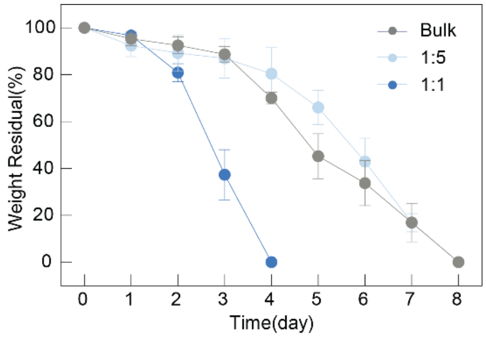

Figure S8. Degradation of 12.5% GelMA bulk hydrogel and heterogeneous hydrogel composites with 1:5 and 1:1 photocrosslinked microgel to bulk volume ratios in PBS.

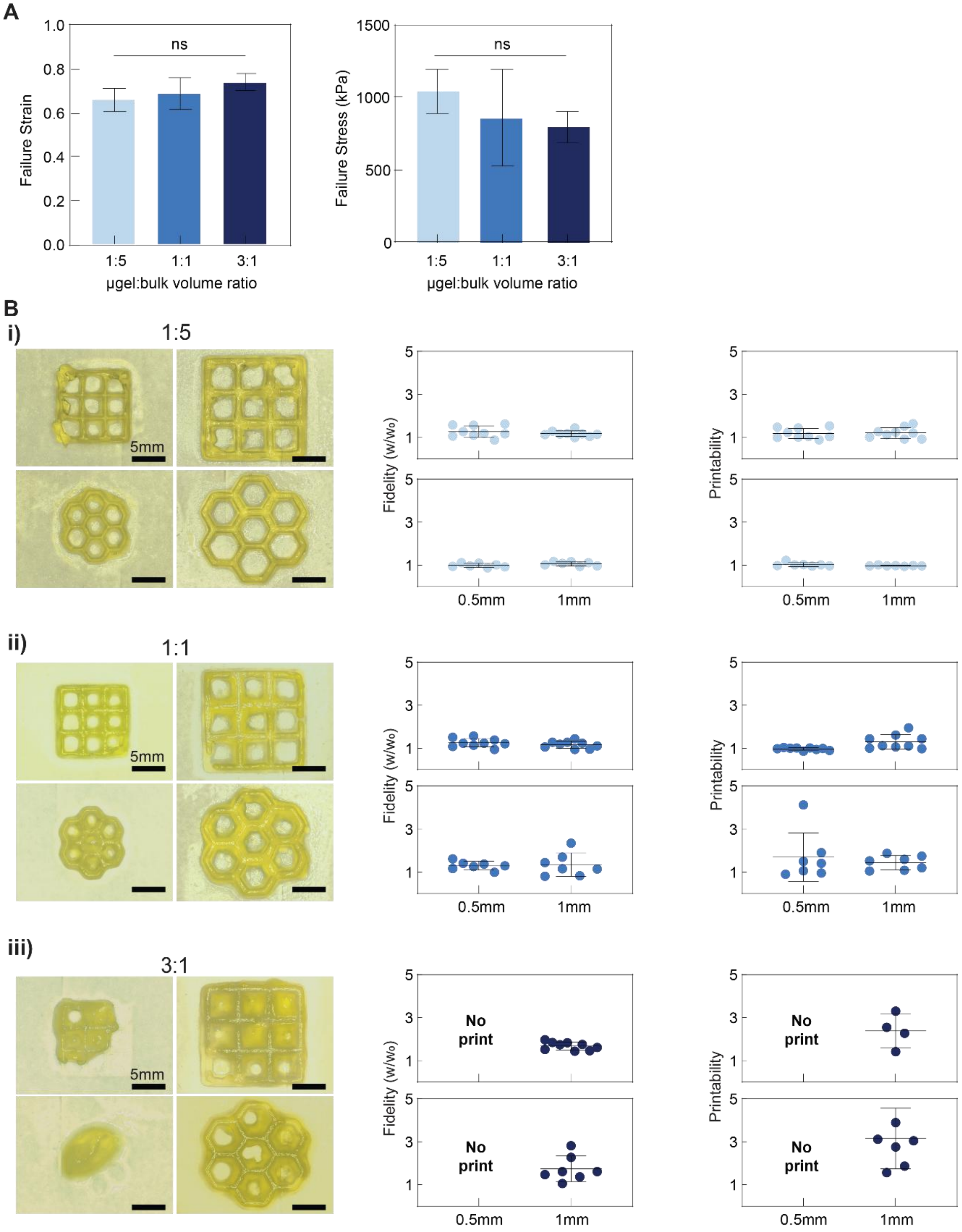

Figure S9. A Compression test of heterogeneous hydrogel composites printed with varying physically crosslinked microgel to bulk volume ratios (n=3): failure strain (left) and failure stress

(right). **B** Printing of composite bioink with varying physically crosslinked microgel to bulk volume ratios (**i)** 1:5, **ii)** 1:1, **iii)** 3:1), including images of printed structures (left), quantification of fidelity (center), and quantification of printability (right). All experiments were performed with 12.5% GelMA for both microgel and bulk concentrations. Images from **Fig. 4E** are reused for fidelity and printability comparison. Statistical analysis performed using a one-way ANOVA, ns = no significance.
